# Efficient Authentication Protocol and Its Application in Resonant Inductive Coupling Wireless Power Transfer Systems

**DOI:** 10.3390/s21248245

**Published:** 2021-12-09

**Authors:** Emmanuel Ahene, Mark Ofori-Oduro, Frimpong Twum, Joojo Walker, Yaw Marfo Missah

**Affiliations:** 1 Department of Computer Science, Kwame Nkrumah University of Science and Technology, PMB, UPO, KNUST, Kumasi, Ghana; ftwum.cos@knust.edu.gh (F.T.); ymissah@gmail.com (Y.M.M.); 2 Department of Electrical and Computer Engineering, Concordia University, 1455 De Maisonneuve Blvd, Montreal, QC H3G IM8, Canada; oduromark91@gmail.com; 3 School of Software Engineering, University of Electronic Science and Technology of China, Chengdu 610051, China; joojokojododzi@gmail.com

**Keywords:** chaos-based cryptography, key exchange, authentication protocol, wireless power transfer

## Abstract

Chaos theory and its extension into cryptography has generated significant applications in industrial mixing, pulse width modulation and in electric compaction. Likewise, it has merited applications in authentication mechanisms for wireless power transfer systems. Wireless power transfer (WPT) via resonant inductive coupling mechanism enables the charging of electronic devices devoid of cords and wires. In practice, the key to certified charging requires the use of an authentication protocol between a transmitter (charger) and receiver (smartphone/some device). Via the protocol, a safe level and appropriate charging power can be harvested from a charger. Devoid of an efficient authentication protocol, a malicious charger may fry the circuit board of a receiver or cause a permanent damage to the device. In this regard, we first propose a chaos-based key exchange authentication protocol and analyze its robustness in terms of security and computational performance. Secondly, we theoretically demonstrate how the protocol can be applied to WPT systems for the purposes of charger to receiver authentication. Finally, we present insightful research problems that are relevant for future research in this paradigm.

## 1. Introduction

In the wireless power transfer (WPT) concept [1], a transmitter device, driven by a source of electric power, produces a time-varying electromagnetic field that transmits power crosswise over space to the receiver device. The receiver device extracts power from the field and then supplies this to an electrical load. Since the emergence of WPT technology, the traditional usage trends of electrical energy have been significantly changing, rendering the use of wires and power cords unattractive and impractical for mobility and large-scale deployments [2]. The diverse forms of WPT are classified as radiative and non-radiative based on their transmission techniques and distance of transmission. In the radiative WPT, power is transmitted over long distances by means of electromagnetic waves such as radio frequency (RF) waves, microwaves or laser beams [3,4]. In contrast, power is transmitted over short distances by means of electromagnetic fields coupling such as inductive, resonant inductive, magnetic or capacitive coupling in the non-radiative WPT [5,6,7]. Both technologies have useful applications in the transfer of energy with differences in power transfer efficiency (PTE). The scope of this paper is limited to non-radiative WPT systems that exist via resonant inductive coupling techniques.

In earlier applications of WPT using inductive power transfer (IPT), designers were faced with the challenge of reduced energy efficiency since the strength of the induced magnetic field decreased with respect to distance. As a remedy, the concept of WPT using resonant inductive coupling was introduced [5]. The inception of resonators with the same frequency in the sources and receiver coil, respectively, ensures that both systems couples magnetically, hence allowing for higher efficiency in energy transfer. This implies that power transfer occurs over an air gap devoid of metal or any material connection. However, when the two objects are far apart, power transfer is achievable via resonating the two coils at the same frequency. Greater power transfer distance is attainable with resonant repeaters between the two components. Until now, WPT using resonant inductive coupling holds much promise for future technology since its range of transmission is the largest range among the other techniques in the non-radiative WPT systems [8]. It has merited applications in biomedical implants, charging portable devices, electric vehicles and smartcards.

Although the resonant inductive coupling technique has multiple benefits, it is associated with unexpected security vulnerabilities. Firstly, the rate of energy harvesting can enormously change due to the sensitivity of energy transmitters (ET) to the surrounding environment (which is the presence of other energy receivers (ER) beside their targeted ER). Secondly, it is possible for an adversarial ET (such as counterfeit wireless chargers) to initiate a launch or an attack that can cause power surges that can fry the ER device’s circuitry [9,10]. The absence of relevant security measures in resonant inductive coupling WPT systems may slow down their rapid adoption in the future. The emphasis of this paper is to provide a key exchange authentication mechanism that can be applied to resonant inductive coupling WPT systems. We leverage the deterministic random-like property of chaos theory to achieve our objective in this paper.

Chaos-based cryptography has generated significant applications in industrial mixing, pulse width modulation and in electric compaction [11,12]. Likewise, it has merited applications in authentication and energy encryption mechanisms [13,14,15] for wireless power transfer systems. In [13,14,15], the authors proposed several energy encryption techniques for resonant inductive coupling WPT systems using chaos-based cryptographic techniques. However, in their approaches, they only achieve confidentiality and they lack authentication. We point out that their approaches do not provide perfect forward secrecy and resistance to replay attacks that are essential security requirements for resonant inductive coupling WPT systems. 

Perfect forward secrecy assures that an adversarial ET does not compromise session keys, which are relevant to generating switching frequencies for both authorized ET and ER, even at the compromise of one party’s private key. Moreover, resistance to replay attack can prevent an unauthorized ER or a malicious device from delaying the process of electric power charging. As a remedy, we propose a key exchange authentication protocol from chaos theory and demonstrate how it can be applied to the realization of a secure WPT system that assures equitable power transfer. Our scheme achieves mutual authentication, perfect forward secrecy, resistance to replay attack and known key security. For simplicity, we redefine “resonant inductive coupling WPT systems” as “WPT systems” and “key exchange authentication protocol” as “authenticated key exchange scheme” in the subsequent sections of the paper.

### 1.1. Related Work

In a concise manner, we present related works pertinent to the WPT system paradigm and the authenticated key exchange scheme (AKE) paradigm.

On WPT systems, Kurs et al. [2] primarily introduced the notion of wireless transmission of power through strongly coupled magnetic resonance. In their work, they experimentally demonstrated the efficiency of non-radiative power transfer over distances up to eight times the radius of a coil with a 40% efficiency in the transfer of 60 watts. Wang et al. [11] accordingly worked on the system structure authorization and principle explanation of WPT by way of strongly coupled magnetic resonances (SCMR). In general, their work analyzes the characters of the multicoil system of SCMR and emphasizes the instructions for designing practical WPT system structures. In [12], Rajiv et al. significantly contributed to the WPT paradigm by proposing the resonant coupling analysis for a two-coil wireless power transfer system. On the other hand, Lee et al. [16] proposed that wireless transmission can be done using a different approach by presenting reflexive field containment in dynamic inductive power transfer systems. In 2009, Cannon et al. [17] proposed magnetic resonant coupling as a potential means for wireless power transfer to multiple small receivers. In summary, these related works and others such as [7,18] focus on the efficiency mode of transfer, the working principles and the circuit topology of WPT systems. Few existing works attempt to address the security issues of WPT systems. In [13] and [14], Zhen et al. proposed the energy encryption technique for wireless power transfer. However, their work does not assure perfect forward secrecy and resistance to replay attack. In [19], genuine chargers are authenticated using public key authentication mechanisms such as elliptic curve cryptography. This approach is meant to overcome the challenge of skewness in received power between ERs and secure the ERs from perceived attacks by counterfeit ETs. By extension, a generic mechanism [20] based on certificateless cryptography has been designed for improving secure WPT systems. However, according to [21], it may be a computationally expensive approach for encrypting the resonant frequency.

AKE schemes allow for two entities to securely communicate over insecure channels with shared keys. After its inception [22] by Diffie and Hellman, several useful AKE schemes [23,24,25,26] have been developed with diverse application perspectives. The adoption of chaos theory into AKE is due to its suitable properties of extreme sensitivity to initial conditions, pseudo-randomness, low computational cost, unpredictability and non-periodicity [27,28,29]. Essentially, the concept of chaos-based AKE [30,31] has shown merit in secure smartcard transactions [32], Internet of Things [33], smart grids [34] and WPT systems [13,14,15]. Several chaos-based AKE schemes [24,35,36,37,38] have been developed over the years. However, most of them may not be directly applicable to achieve a secure WPT system. For instance, the schemes in [35,38] are computationally expensive and may not be fitting for WPT devices. In contrast, recent works such as [13,14,15] are applicable to WPT systems but they do not provide perfect forward secrecy and resistance to replay attack. In this perspective, we put forward an efficient chaos-based AKE (CBAKE) scheme that can be applied to WPT systems by extension and can assure secure and equitable power transfer.

### 1.2. Organization

The rest of the paper is organized as follows: In Section 2, preliminary concepts employed in the paper are presented. In Section 3, the concrete construction of the proposed scheme and its security analysis are presented. We evaluate the performance of our scheme and compare it to other chaos-based AKE schemes in Section 4. In Section 5, we show how our scheme can be applied to realize a secure WPT system and also compare our proposed scheme to other existing WPT schemes in terms of functionality. In Section 6, we present the open research questions that can extend research in this domain. Finally, we conclude this paper in Section 7.

## 2. Preliminaries

### 2.1. Wireless Power Transfer Scenario

**Figure 1:**
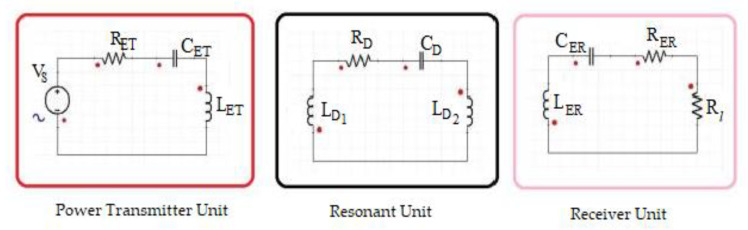
Wireless power transfer (WPT) system basic circuit.

Transfer of energy from the transmitter to the receiver is achieved by ensuring that both systems are resonating at the same frequency [39,40,41]. We give a further explanation using Figure 1. According to Figure 1, it is obvious that the basic circuit of a WPT system is made up of three fundamental units, namely the transmitter, resonator and receiver. We denote $V_{S}$ as the source voltage, $R_{ET}$ as the resistance, $C_{ET}$ as the capacitance and $L_{ET}$ as the inductance of the transmitter. $C_{D}{,\;R}_{D}$, and $L_{D1}/L_{D2}$ are the capacitance, resistance and inductance of the resonator, respectively, while $C_{ER},\;{\;R}_{ER}$ and $L_{ER}$ are the capacitance, resistance and inductance of the receiver. Finally, $R_{l}$ is identified as the load resistance. The switching frequencies [42,43] of the transmitter $\omega_{ET}$ resonator $\omega_{D}$ and receiver $\omega_{ER}$ are mathematically defined as; $\omega_{ET}=\frac{1}{\sqrt{C_{ET}L_{ET}}}{,\;\omega }_{D}=\frac{1}{\sqrt{C_{D}L_{D}}}{,\;\omega }_{ER}=\frac{1}{\sqrt{C_{ER}L_{ER}}}$ Hence, to obtain maximum power transfer from the transmitter to the receiver, all units of the circuit must reach the same frequency of resonance as shown in Equation (1). This can be achieved by simultaneously adjusting the capacitance on the transmitter’s side as well as the receiver’s side. The resonance coil is necessary for the increase of the transmission distance between the transmitter and the receiver:

(1) $$\omega_{ET\;}{=\omega }_{ER}{=\omega }_{ED}$$

### 2.2. Chebyshev Chaotic Map

The Chebyshev polynomial [44,45] of degree *n* is defined as:

$$T_{n}\left(x\right)=cos(n*arccos\left(x\right))(-1\;\leq \;x\;\leq \;1)$$

The recurrent formulas are:

$$T_{0}\left(x\right){=1,\;T}_{1}\left(x\right){=x,\;T}_{2}\left(x\right){=2x}^{2}-1,\;\ldots ,$$

$$T_{n+1}\left(x\right)=2xT_{n}\left(x\right)\;{-\;T}_{n-1}\left(x\right),\;n=1,\;2,\ldots .$$

Chebyshev polynomial also exhibits the following properties:

(1) Semi-group property

$$\begin{array}{cc} T_{r}\left(T_{s}\left(x\right)\right) & =cos(r*arccos(cos(s*arccos(x))))\\ & =cos\left(rs*arccos\left(x\right)\right)\\ & =T_{sr}\left(x\right)\\ & {=T}_{s}{(T}_{r}\left(x\right))\\ & ∴{\;T}_{r}\left(T_{s}\left(x\right)\right){=T}_{sr}\left(x\right){=T}_{s}\left(T_{r}\left(x\right)\right)\forall \;s,r\;\in {\;Z}^{+} \end{array}$$

(2) Chaotic property when *n >* 1, Chebyshev polynomial map $T_{n}:\left[-1,\;1\right]\to \left[-1,\;1\right]$ of degree *n* is a chaotic map with its invariant density:

$$f^{*}\left(x\right)=\frac{1}{\sqrt[{\pi }]{1-x^{2}}}$$

For the Lyapunov exponent, λ=lnn > 0.

### 2.3. Hard Problem

Our scheme is based on three hard problems, namely: the hardness of the quadratic residue assumption and the two hard problems associated with the semi-group property of the Chebyshev polynomial, namely the chaotic-based discrete logarithm (CDL) problem and the chaotic-based Diffie–Hellman (CDH) problem. These hard problems are assumed to be infeasible to solve if one is not aware of some specific parameters. In other words, no polynomial time algorithm has been found to solve such problems. We give the details of the problems as follows:

(1) Quadratic Residue Assumption: Given *p* and *q* as two large primes and *n* = *p* ∗ *q*. Let the symbol ${QR}_{n}$ denote the set of all quadratic residues in [1, *n* − 1]. If ${y=x}^{2}\;mod\;n$ has a solution, i.e., ∃ a square root for *y*, then *y* is named as a quadratic residue modulo *n* where $y\;\in {\;QR}_{n}$. To find *x* satisfying ${y=x}^{2}\;mod\;n$ when *p* and *q* are unknown is computationally intractable since no polynomial algorithm has been found to solve the factoring problem.
(2) Chaotic-based Discrete Logarithm (CDL) Problem: Given the variable *x* and the result *y*, it is infeasible to find the integer *n*, such that $T_{n}\left(x\right)\;\equiv \;y\;mod\;p$
(3) Chaotic-based Diffie–Hellman (CDH) Problem: Given the variable *x*, $T_{n}\left(x\right)\;mod\;p$ and $T_{m}\left(x\right)\;mod\;p$, it is infeasible to compute $T_{nm}\left(x\right)\;mod\;p$ without knowing *n* or *m*.

## 3. A Chaos-Based Authentication Key Exchange Scheme

In this section, we outline the concrete construction of the CBAKE scheme.

Phase 1: System Initialization

With a security parameter *λ*, the energy transmitter ET first generates large primes *p* and *q* and computes *n = p* ∗ *q.* Here, *p* and *q* are kept as secret keys, whereas *n* is published by the ET. Additionally, the ET publishes two hash functions $H_{1}{:\{0,1\}}^{*}\to {\left\{0,1\right\}}^{\lambda }$ and $H_{2\;}{:\{0,1\}}^{*}\to (-\infty ,+\infty )$.

Phase 2: Authentication and Key Exchange

▪ First, the ER chooses integers *r* and *y* at random and computes *x*${=H}_{1}\left(y\right){,\;T}_{r}\left(x\right){=r}_{pub}{,\;k=y*r}_{pub}{,\;\mu =(k||PW),\;r=\mu }^{2}{\;mod\;n,\;EID}_{ER}{=U}_{ER}⊕H_{2}\left(\mu \right){\;\mathrm{and}\;UAuth}_{ER\;}=$ ${\;H}_{2}\left({\mu ,\;T}_{r}\left(x\right){,\;T}_{1}{,\;EID}_{ER}\right).$ Here, $T_{1}$ is the initial timestamp. The ER sends $C_{1}{=\{UAuth}_{ER}{,\;r,\;T}_{r}\left(x\right){,\;T}_{1}{,\;EID}_{ER}\}$ to the ET.
▪ Given *C*_1_, the ET validates whether $T_{2}-T_{1}\;\leq \;△T$ is true or not. Here, $T_{2}$ is the ET’s current timestamp. Upon successful verification, the Chinese remainder theorem is used to solve *R* using *p* and *q* to get $\mu_{1},\;\mu_{2},\;\mu_{3},\;\mu_{4}$ and then the ET determines whether $\mu^{\prime }=(k^{\prime }||PW^{\prime }\;)$ by verifying whether ${UAuth}_{ER}{=H}_{2}{(\mu }_{i}{,\;T}_{r}\left(x\right){,\;T}_{2}{,\;EID}_{ER})$ for *i =* 1, 2, 3, 4. Subsequently, the ET computes $U_{ER}{=EID}_{ER}⊕H_{2}{(\mu }^{\prime })$ and validates whether ${PW}^{\prime }=PW$ is right or not. If true, the ET successfully authenticates the ER and selects a random integer *s* and computes ${x=H}_{1}{(y}^{\prime })$, $T_{s}\left(x\right),\;\gamma =H_{2}{(T}_{r}\left(x\right){,T}_{s}\left(x\right){,T}_{sr}\left(x\right))$, ${UAuth}_{ET}{=H}_{2}{(\gamma ,\;PW,U}_{ET}{,U}_{ER}{,T}_{2})$. The ET then sends $C_{2}{=\{UAuth}_{ET}{,U}_{ET}{,T}_{s}\left(x\right){,T}_{2}\}$ to the ER.
▪ It is verified whether $T_{3}-T_{2}\leq \;△T$ is true or not once *C_2_* is received by the ER. Note that $T_{3}$ is the current timestamp here. The ER then computes $\gamma^{\prime }=H_{2}{(T}_{r}\left(x\right){,T}_{s}\left(x\right){,T}_{sr}\left(x\right))$ and validates the rightness of ${UAuth}_{ET}{=H}_{2}{(\gamma }^{\prime }{,PW,U}_{ET}{,U}_{ER}{,T}_{2})$. Once verified as right, the ER successfully authenticates the ET; otherwise, the ER aborts this request. Now, the ER and the ET possess $\gamma =H_{2}{(T}_{r}\left(x\right){,T}_{s}\left(x\right){,T}_{rs}\left(x\right))$. Thus, γ is the shared session key, which is relevant for computing the switching frequency.

### Security Analysis of the CBAKE Scheme

In this subsection, we analyze the proposed chaos-based authenticated key exchange scheme in terms of its security and performance. Our proposed scheme is secured in terms of mutual authentication, contribution property of key agreement, private key security, perfect forward secrecy, resistance to password guessing attack, user anonymity, known key security and resistance to replay attack.

1. Mutual Authentication: In the proposed scheme, the ET authenticates the ER by verifying $H_{2}\left({EID}_{ER}{,\;\mu }_{i}{,\;T}_{r}\left(x\right){,\;T}_{1}\right){=UAuth}_{ER}$ and ${PW}^{\prime }=PW$. Subsequently, the ER authenticates the ET by verifying $H_{2}{(\gamma }^{\prime }{,PW,U}_{ET}{,U}_{ER}{)=UAuth}_{ET}$ as stated in the third step, where $\gamma^{\prime }=H_{2}{(T}_{r}\left(x\right){,T}_{s}\left(x\right){,T}_{s}{(T}_{r}\left(x\right)))$. Hence, the proposed scheme has mutual authentication capability.
2. Resistance to Replay Attack: The proposed scheme guarantees the freshness of the key due to the timestamps being utilized. These can be seen as follows: *T*_1_ in *C*_1_, *T*_2_ in *C*_2_ and *T*_3_ in *C*_3_. Therefore, our proposed scheme prevents replaying attacks.
3. Contribution Property of Key Agreement: In the proposed scheme, the chaotic session key is $\gamma =H_{2}{(T}_{r}\left(x\right){,T}_{s}\left(x\right){,T}_{rs}\left(x\right))$. In the process, no party is able to determine the session key alone since *s* and *r* are random numbers secretly generated by the power transmitter and the receiver, respectively. Notably, the proposed scheme satisfies the contribution feature of the key agreement.
4. Private Key Security: Given *T*(*·*), *T_r_*(*x*) and *T_s_*(*x*). *T_rs_*(*x*) = *T_r_*(*T_s_*(*x*)) = *T_s_*(*T_r_*(*x*)), the session key $\gamma =H_{2}{(T}_{r}\left(x\right){,T}_{s}\left(x\right){,T}_{rs}\left(x\right))$ cannot be calculated if *r*, *s* and *x* remain unknown, due to the chaotic maps Diffie–Hellman problem [46]. Therefore, the session key cannot be derived by an unauthorized user in our proposed CBAKE scheme.
5. Known Key Security: The session key $\gamma =H_{2}{(T}_{r}\left(x\right){,T}_{s}\left(x\right){,T}_{rs}\left(x\right))$ generated in distinct rounds are not dependent on each other due to the fact that *r*, *s* and *x* are chosen randomly by the ER and the ET, respectively, and, in the scheme executions, they are independent of each other. Hence, the proposed scheme achieves the known-key security.
6. Perfect Forward Secrecy: In our scheme, a false password *PW* will not result in any previous session key $\gamma =H_{2}{(T}_{r}\left(x\right){,T}_{s}\left(x\right){,T}_{rs}\left(x\right))$ since the short-lived numbers *r*, *s* and *x* are picked randomly and independent among the executions of the scheme’s algorithms. More specifically, the proposed scheme has perfect forward secrecy. However, an attacker can use the strategy of Bergamo et al. [44] to realize the secret key *y* and derive previous session keys $\gamma =H_{2}{(T}_{r}\left(x\right){,T}_{s}\left(x\right){,T}_{rs}\left(x\right))$, where ${x=H}_{1}\left(y\right)$ if the private keys *p* and *q* of *T* are known.
7. Resistance to Password Guessing Attack: For ${V=H}_{2}{(EID}_{ER}{,\;\mu ,\;T}_{r}\left(x\right))$, where μ=(k||PW), ${UAuth}_{ET}{=H}_{2}{(\gamma ,PW,U}_{ET}{,U}_{ER})$ involve password related information. Even though some of the messages are revealed, *PW* cannot be obtained due to the hash function $H_{2}(\cdot )$, which has a one-way property. Moreover, *PW* is protected by the secret value *k*. Additionally, there exists no information that can aid an attacker to directly confirm the authenticity of the guessed passwords. In this way, offline password guessing attacks fail with respect to our proposed scheme.
8. User Anonymity: The temporary identity ${EID}_{ER}{=U}_{ER}⊕H_{2}\left(\mu \right)$, where μ=(k||PW) and *k* represent a random secret generated by the ER is not dependent on scheme executions. Therefore, ${ID}_{ER}$ cannot be obtained from ${EID}_{ER}$ when *k*, *PW* and likewise ${ID}_{ET}$ are unknown. Moreover, due to the quadratic residue assumption, one cannot decipher μ from *R* if the power transmitter’s secret keys *p* and *q* are not known, where ${R=\mu }^{2}\;mod\;n$. In addition, $U_{ER}$ and $U_{ET}$ cannot be generated from ${UAuth}_{ER}{=H}_{2}{(\gamma ,\;U}_{ER}{,U}_{ET})$, ${UAuth}_{ET}{=H}_{2}{(\gamma ,PW\;U}_{ER}{,U}_{ET})$ because of the inherent one-way property of the hash function. Hence, our proposed CBAKE scheme achieves the user anonymity feature.

## 4. Performance Analysis

In this section, we present a performance analysis of our proposed CBAKE scheme in relation to other chaos-based schemes. We highlight the computation cost of each of the schemes and also compare some of the significant properties that these schemes possess. The results from our analysis and the corresponding notations used for the analysis with their meanings are presented in Table 1 and Table 2, respectively. In Table 1, we follow the experimental results in [35] as a standard to evaluate all the schemes under comparison. Researchers in [35] report that when using the PBC library on an Ubuntu 12.04.1 32 bit operating system, with 2.4 GHz CPU and 2.0 GB RAM, the estimated running times for various cryptographic operations are as follows: the time of the hash-based operation is 0.00058 s, the symmetric encryption or decryption is 0.0086 s, the modular squaring operation, modular square root operation, elliptic curve scalar multiplication and Chebyshev polynomial operation are 0.01018 s, 0.00987 s, 0.063165 s and 0.02104 s, respectively. They considered the XOR operation cost as negligible in their analysis. As shown in Table 1, we begin our comparison with the various computational cost evaluated. 

The schemes presented in [35,38,47] are computationally expensive compared to our proposed CBAKE scheme and the other schemes making them inefficient for practical applications. Moreover, we point out that scheme [38] exhibits some weaknesses with regards to resisting possible attacks. On the other hand, chaotic maps-based schemes [48,49,50,51] exhibit low computations and are therefore ideal for practical applications. However, due to the fact that they do not support user anonymity and are also weak in resisting possible attacks, they will not be expedient to use in applications such as WPT systems. All the schemes presented in Table 1 except [51] support perfect forward secrecy, which is an important feature for practical applications such as WPT systems. The researchers in [52] revealed that scheme [51] lacks this important feature of perfect forward secrecy. Again, schemes [53,54] have lower computational cost than our proposed scheme. This is mainly due to the fact that quadratic residues are employed in protecting a user’s password in our proposed CBAKE scheme. In our scheme, one modular squaring operation is needed by an ER, while the ET needs one squaring root solving operation. Conversely, it has been shown in [55,56] that a modular squaring operation is equivalent to a few hundred gates. The implementation of SHA-1, MD5 and the universal hash function requires 20 K gates, 16 K gates and 1.7 K gates, respectively. 

Hence, it is worth mentioning that the ET’s efficiency in CBAKE is unaffected by the modular squaring operation. Additionally, no symmetric encryption/decryption operations are carried out by schemes [40,48,50,51,52,53,54] and CBAKE so they achieve lower computational costs from a user perspective. Moreover, CBAKE [ has significant security properties than other related schemes. Furthermore, to protect a weak password, most related schemes employ extra devices, such as smartcards, to store their long-term secret key; only our CBAKE scheme and [48] do not employ smartcards. In our proposed CBAKE scheme, a party only stores their own password and does not need an extra device for storing a long-term secret key.

## 5. Application to WPT System

**Figure 2:**
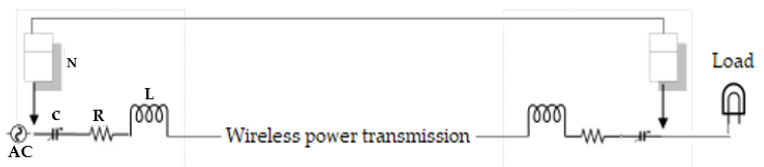
System energy encryption architecture.

**Figure 3:**
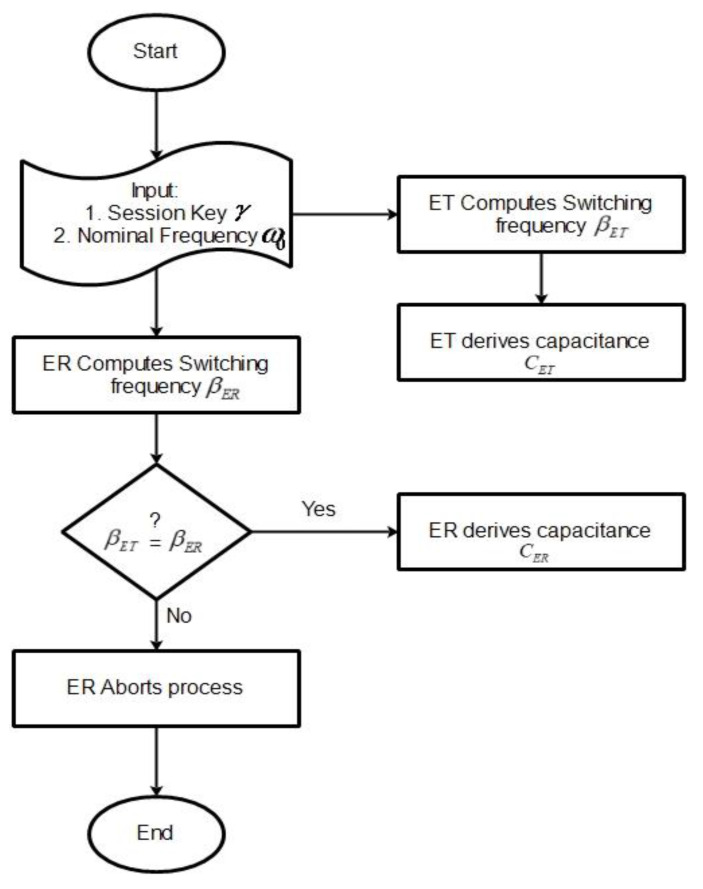
Illustration of the process of encryption and decryption.

In this section, we only demonstrate theoretically the application of CBAKE scheme to a WPT system. Rigorous implementation of the system is out of the scope of the paper. As shown in Figure 2, the system comprises a transmitter device whose source of power is obtained from the main power line. The transmitter device (ET) converts the power to an electromagnetic field, which can be received by one or more receiver devices via resonant inductive coupling. The receiver device (ER) receives power and then converts it back to a direct electric current (DC), which is utilized by the electrical load. Both the transmitter and receiver circuit comprise a resistor, inductor, variable capacitor and a processing entity or node. Essentially, a judicious regulation of the working frequency in the energy transmitter and energy receiver circuits determines the performance of power flow. In other words, power transfer is efficient based on the optimal switching frequency. Subsequently, we refer to the transmitter device as a wireless charger pad (WCP) and the receiver device as a mobile phone. To ensure an efficient and secured wireless power flow, the wireless charger pad establishes trust with the mobile phone by running the CBAKE scheme. The WCP controls the variable capacitor using its obtained chaotic session key. In this way, the frequency of the WCP is also regulated. The mobile phone can only receive power, when, accordingly, its frequency is simultaneously regulated by the same chaotic session key. It is obvious that, once the ephemeral session key is unknown, the usage of the transferred wireless power would be undesirable to other unauthorized receiving devices. We define the process whereby the wireless charger pad and the mobile phone undertakes internal computations and regulations to resonate at the same frequency using their ephemeral chaotic session keys as energy encryption and decryption, respectively. The detailed process for the encryption and decryption is depicted in the flowchart in Figure 3 and mathematically deduced as follows.

Encrypt $\left({\gamma ,\;\omega }_{0}\right)\to \left(\beta_{ET}{,\;C}_{ET}\right):$ The encrypt algorithm is run by the ET. The ET takes as input the chaotic session key $\gamma =H_{2}{(T}_{r}\left(x\right){,T}_{s}\left(x\right){,T}_{rs}\left(x\right))$ and the nominal frequency $\omega_{0}$ and outputs a switching frequency $\beta_{ET}{=\gamma \omega }_{0}$ and capacitance *C_ET_*. *C_ET_* is computed as follows: if the chaotic session key for both parties is the Chebyshev polynomial $\gamma =H_{2}{(T}_{r}\left(x\right){,T}_{s}\left(x\right){,T}_{rs}\left(x\right))$ then the ET can compute a switching frequency as $\beta_{ET}{=\gamma }_{i\;}\omega_{0},\forall \;i\;>\;0.$ Assuming $\omega_{ET}=\beta_{ET}$ then the ET can compute *C_ET_* as:

(2) $$\omega_{ET}=\frac{1}{\sqrt{L_{ET}C_{ET}}};\;{\gamma \omega }_{0}=\frac{1}{\sqrt{L_{ET}C_{ET}}};\;C_{ET}=\frac{1}{\sqrt{\gamma^{2}\omega_{0}^{2}L_{ET}}}$$

Decrypt $\left({\gamma ,\;\omega }_{0},\;C_{ET}\right)\to \left(\beta_{ER},C_{D}{,\;C}_{ET},\;\right):$ The receptor ER computes the switching frequency $\beta_{ER}$ using their chaotic session key and a nominal frequency $\omega_{0}$ as input. Assuming $\omega_{ER}=\beta_{ER}$ and, if $\beta_{ER}{=\beta }_{ET}$*,* then the ER can derive its capacitance as *C_ER_*. *C_ER_* is computed as follows:

(3) $$C_{D}=\frac{1}{\gamma^{2}\omega_{0}^{2}L_{D}}{\;;\;C}_{ER}=\frac{1}{\gamma^{2}\omega_{0}^{2}L_{ER}};.$$

The continuous variation of the capacitance value enhances the security performance of the WPT system. In this work, we achieve the continuous variation using the ephemeral session key. Since both ends can obtain the optimal switching frequency, a secure power transfer is assured. 

### Functionality Comparison

Now, to point out the significance of our work, we compare the basic properties of the following encryption schemes [13,14,15] for WPT systems to our proposed CBAKE scheme. The CBAKE scheme supports mutual authentication but [13,14,15] do not achieve mutual authentication. This property prevents an adversarial ER from harnessing power. Moreover, our scheme is resistant to replay attack. This important property is missing in schemes [13,14,15]. Additionally, we indicate that a WPT system supporting perfect forward secrecy can ensure that an adversarial ET does not compromise session keys. This is significant when generating switching frequencies for both authorized ET and ER even in instances where one party’s session key is compromised. Yet again, the perfect forward secrecy feature is absent in schemes [13,14,15].

## 6. Open Research Problems

In this section, we enumerate some open research questions that are targeted at ensuring the expansion of this research field. To begin with, considering the resource constrained devices involved in secure WPT systems, a desirable requirement would be to develop and deploy encryption schemes that are lightweight. It is therefore significant to design schemes that have a low computational cost. Furthermore, it is important to design schemes that ensure that there is low power consumption at the nodes in each circuit system. Additionally, since it is significant to achieve fast, stable and secure wireless power transfers, it would be expedient for this research community to investigate the time scale over which the switching frequency varies. It is worth noting that an intelligent attacker unaware of the key can employ small and slow manipulations to the frequency to extract significant amounts of energy. They achieve this by dynamically changing the frequency so as to maximize received energy. Frequency changes that are fast and heavy can result in secondary effects such as differences in frequency ranges of signal components. More research can be carried out on the effects of these factors and more significantly into building a trade-off between them. Finally, it would be interesting to consider how protocols supporting standard wireless communication technologies such as Bluetooth Low Energy and near-field communication can be adapted into the application of resonant inductive coupling WPT systems.

## 7. Conclusions

In this paper, we have proposed a new chaos-based authenticated key exchange scheme. We have further demonstrated how the scheme can be applied to WPT systems. The proposed scheme primarily establishes trust and exchanges a common session key via an authenticated key exchange protocol, between the energy transmitter and energy receiver. In spite of the fact that our scheme requires extra computation compared to some existing schemes, we point out that our scheme does not need additional devices such as a smartcard for storing long-term passwords, a feature which is pertinent to WPT systems. Our proposed scheme is highly feasible for other practical applications. 

## Figures and Tables

**Table 1:** Performance evaluation.

Schemes	Computation Cost	Computation Time in Seconds	F1	F2	F3	F4
[35]	49T_H_ + 10T_C_ + 2T_S_	0.25602	No	Yes	Strong	Yes
[38]	43T_H_ + 10T_C_	0.23534	No	Yes	Weak	Yes
[47]	18T_H_ + 10T_C_	0.22084	No	No	Weak	Yes
[48]	7T_H_ + 4T_C_	0.0882	Yes	No	Weak	Yes
[49]	5T_H_ + 6T_C_ + 5T_S_	0.17214	No	No	Weak	Yes
[50]	12T_H_ + 4T_C_	0.09112	No	No	Weak	Yes
[51]	5T_H_ + 4T_C_ + 5T_S_	0.13006	No	No	Weak	No
[53]	21T_H_ + 6T_C_	0.13842	No	Yes	Strong	Yes
[54]	9T_H_ + 4T_C_	0.08938	No	Yes	Strong	Yes
Ours	13T_H_ + 4T_C_ + T_SQ_ + T_SR_	0.20337	Yes	Yes	Strong	Yes

**Table 2:** Meanings of symbols used for performance evaluation.

Notation	Meaning
F1	Non-usage of extra device such as smartcard
F2	Supports user anonymity
F3	Resistance to possible attacks
F4	Supports perfect forward secrecy
TH	Time for executing a hash function
TC	Time for executing a chaotic map operation
TS	Time for executing a symmetric encryption or decryption operation
TSQ	Time for executing a squaring operation
TSR	Time for executing a square root operation

## Data Availability

Not Applicable.
